# Elevating Voices, Addressing Depression, Toxic Stress, and Equity Through Group Prenatal Care: A Pilot Study

**DOI:** 10.1089/heq.2023.0160

**Published:** 2024-01-29

**Authors:** Shannon N. Lenze, Kelly McKay-Gist, Rachel Paul, Melissa Tepe, Katherine Mathews, Sara Kornfield, Cheron Phillips, Richelle Smith, Amanda Stoermer, Ebony B. Carter

**Affiliations:** ^1^ Department of Psychiatry, Washington University School of Medicine, St. Louis, Missouri, USA.; ^2^ St. Louis Integrated Health Network, St. Louis, Missouri, USA.; ^3^ Department of Obstetrics and Gynecology, Washington University School of Medicine, St. Louis, Missouri, USA.; ^4^ Affinia Healthcare, St. Louis, Missouri, USA.; ^5^ SSM Health St. Mary's and Department of Obstetrics, Gynecology, and Women's Health, St. Louis University, St. Louis, Missouri, USA.; ^6^ Center for Women's Behavioral Wellness, Department of Psychiatry, Perelman School of Medicine at the University of Pennsylvania, Philadelphia, Pennsylvania, USA.

**Keywords:** mental health, health equity, group prenatal care, patient-centered outcomes research

## Abstract

**Introduction:**

Elevating Voices, Addressing Depression, Toxic Stress and Equity (EleVATE) is a group prenatal care (GC) model designed to improve pregnancy outcomes and promote health equity for Black birthing people. This article outlines the foundational community-engaged process to develop EleVATE GC and pilot study results.

**Methods:**

We used community-based participatory research principles and the Ferguson Commission Report to guide creation of EleVATE GC. The intervention, designed by and for Black birthing people, centers trauma-informed care, antiracism, and integrates behavioral health strategies into group prenatal care to address unmet mental health needs. Using a convenience sample of patients seeking care at one of three safety-net health care sites, we compared preterm birth, small for gestational age, depression scores, and other pregnancy outcomes between patients in individual care (IC), CenteringPregnancy™ (CP), and EleVATE GC.

**Results:**

Forty-eight patients enrolled in the study (*n*=11 IC; *n*=14 CP; *n*=23 EleVATE GC) and 86% self-identified as Black. Patients participating in group prenatal care (EleVATE GC or CP) were significantly less likely to experience a preterm birth <34 weeks. Rates of small for gestational age, preterm birth <37 weeks, depression scores, and other pregnancy outcomes were similar across groups. Participants in CP and EleVATE GC were more likely to attend their postpartum visit and breastfeed at hospital discharge than those in IC.

**Discussion:**

Our findings model a systematic approach to design a feasible, patient-centered, community-based, trauma-informed, antiracist intervention. Further study is needed to determine whether EleVATE GC improves perinatal outcomes and promotes health equity.

## Introduction

The burden of perinatal morbidity and mortality is not borne fairly nor equitably across the United States. Black women and their offspring are at significantly higher risk for adverse events than White women, including preterm birth,^1^ cardiovascular disease,^2^ and low birthweight.^3^ These disparities persist across all socioeconomic strata and are present after accounting for prenatal care, parity, age, education, marital status, and substance use disorder.^4^ There are several potential sources of perinatal health disparities,^5^ including exposure to racism that significantly increases the risk of chronic psychosocial stress, adversity, and traumatic life events.^6^

Decades of literature have consistently linked depression symptoms, pregnancy-related anxiety, perceived stress, stressful life events, early adversity, and the experience of racial discrimination with adverse pregnancy and infant outcomes.^7–9^ Furthermore, Missouri state-level data indicate that mental health conditions are the leading contributor to maternal mortality.^10,11^ Differences in access to mental health interventions disproportionately affect minoritized populations.^12–14^ Thus, it is imperative to improve models of perinatal care in ways that can address these social determinants of health and root causes of health inequities.

One such promising model is group prenatal care, which has gained increasing attention in recent years. Group prenatal care was developed to provide increased prenatal education and social support, factors thought to be important for improving pregnancy outcomes.^15^ Group prenatal care is an efficient and effective way to provide prenatal care.^16^ These models generally include small groups of pregnant patients of similar gestational ages meeting with a clinician and cofacilitator (often a medical assistant, health educator, or social worker) for ∼10 sessions to discuss topics relevant to pregnancy and the postpartum period in a fun interactive format using adult-learning principles.^17,18^

Early studies of CenteringPregnancy™ (CP), a commonly practiced form of group prenatal care in the United States, suggested significant reductions in preterm birth compared with traditional care.^19–21^ These findings were not replicated in our systematic review and meta-analysis.^22^ However, when the meta-analysis data were disaggregated by race, Black women participating in high-quality group prenatal care studies had a significantly lower risk of preterm birth than Black women in individual care (IC) (pooled risk ratio, 0.55; 95% confidence interval, 0.34–0.88).^20,22,23^ These findings suggest that groups at highest risk for adverse pregnancy outcomes may preferentially benefit from group prenatal care interventions and warrant further study.

In addition to the potential benefits for pregnancy outcomes, group prenatal care may also be effective in reducing the burden of perinatal depression and other psychosocial stressors.^24–31^ A prospective cohort study of 248 patients choosing group or individual prenatal care reported patients with high baseline stress, or few personal coping resources, who participated in group care had better psychosocial outcomes and reduced rates of postpartum depression than high-stress/poor coping patients in IC.^26^

Felder et al. found that women randomized to an enhanced form of CP enriched with content for HIV prevention reported better self-efficacy, better interpersonal communication, and less depression than those in IC.^29^ Increases in social support from shared medical appointments might be a contributing factor to these benefits.^32^

We formed a collaborative—including former patients turned community collaborators with lived expertise, community-based organizations, practicing clinicians, and academic researchers committed to increasing racial equity in perinatal outcomes and community-based participatory research (CBPR)—to address the persistently high rates of adverse pregnancy outcomes in St. Louis, Missouri, particularly among historically marginalized communities. In this article, we describe the EleVATE Collaborative's formation, the process we used to develop our group prenatal care intervention, Elevating Voices, Addressing Depression, Toxic Stress, and Equity in Group Prenatal Care (EleVATE GC), and results from a pilot feasibility study conducted at three safety-net health care clinics in St. Louis, Missouri.

## Methods

### Phase 1: Collaborative and curriculum development

The first meeting of the collaborative occurred in the summer of 2016 in the aftermath of the killing of Michael Brown in our community. Attendees included clinicians from each of the group prenatal care sites in St. Louis and were convened by the St. Louis Integrated Health Network (IHN). The IHN works through collaboration and partnership, to achieve quality, accessible, and affordable health care services for all residents with an emphasis on the medically underserved.

Core values agreed upon in the first meeting included (1) promoting equitable pregnancy outcomes, (2) breaking down systemic racism, and (3) patients' active leadership in the collaborative. The first meeting was the only meeting of the collaborative that did not include patients. Six community collaborators with lived experience in pregnancy, parenting, and participation in group prenatal care served as an integral part of the interdisciplinary team and were paid for their time and expertise. Community collaborators represented the voice and interests of patients, directed the programmatic vision, and served key leadership roles on each of the committees.

We focused our efforts on group prenatal care as an evidence-based method to reduce health disparities in reproductive health outcomes, especially for Black patients who comprised the majority of the patient population at the clinical sites.^32^ The collaborative structure consisted of three interconnected committees: (1) a steering committee providing guidance and direction on programmatic strategies, ensuring equity was a central focus of the work, and forming cohesion among the participating health care institutions that were traditionally competitors; (2) a curriculum committee developing the integrated EleVATE group prenatal care curriculum; and (3) an evaluation committee committed to CBPR principles and providing guidance on equitable research methods, measures, and working closely with community members to define meaningful outcomes and source data points important to them.^33,34^

### EleVATE GC curriculum

After reviewing extant group prenatal care models, the curriculum committee saw the need to tailor an intervention to meet the specific needs of Black pregnant patients by explicitly integrating connections between health promotion, racial equity, social determinants of health, and behavioral health. The committee paid particular attention to addressing stress, trauma, harm reduction, and resilience throughout the curriculum. The resulting EleVATE GC is a 10-session (2 h per session) group prenatal care model following the recommended prenatal visit schedule^30^ and includes common discussion topics about pregnancy and infant care (Table 1).

**Table 1.** Elevating Voices, Addressing Depression, Toxic Stress and Equity Group Care Curriculum

Session	Ice breaker	Welcome and mindfulness activity	Pregnancy topic	Self-care topic	Behavioral topic	Closing: Check-out question
1	Common ground	Belly breathing	What I eat	What is self-care?	Mind, body, and behavior connection	What does healthy look like now and in the future?
2	Name game	Mindful eating	Common discomforts during pregnancy	Self-care bank	Finding calmness	What are my pregnancy expectations now and in the future?
3	Dyad intro	R.A.I.N.	Thinking about feeding my baby	What does self-care look like in every part of my life?	Stop, breathe, think	What does my family look like now and in the future?
4	Family tree	Body scan	Sexual decision making	How do I advocate for myself?	Finding calmness	What does my support look like now and in the future?
5	Goal check-in	Belly breathing	Preterm labor and labor	Check in with kindness	Informed decision making	How do I prepare for labor now and in the future?
6	What is your theme song?	Positive self-talk	Labor decisions and birth experience	How do I take care of myself during labor?	Mind, body, and behavior connection and labor	How do I prepare for my baby now and in the future?
7	Beach ball with questions	Mindful listening	Baby and my first days	How will I take care of myself when baby comes home?	Stop, breathe, think, and baby's first days	How do I bond with my baby now and in the future?
8	Guess the fun fact	R.A.I.N.	Baby blues and postpartum	How do I advocate for myself if I experience postpartum depression?	Finding calmness	What support do I need when baby comes home now and in the future?
9	A poem about me	Mindful eating	Newborn care	Emotional wellness check-ins	Finding calmness	How will I parent now and in the future?

R.A.I.N., Recognize, Acknowledge, Investigate, Non-Identify.

Important elements of the CP model, such as brief individual prenatal examinations with a clinician, attendance of support persons, and self-measurement of blood pressure and weight, are also included in EleVATE GC. The curriculum centers antioppressive and trauma-informed values and principles, behavioral health integration, reproductive justice, and the patient workbook includes pictures that are representative of the families receiving care at participating sites.^34–36^ Community collaborators' leadership and role as cocreators, along with obstetric and behavioral health clinicians, assured the curriculum was clear, coherent, culturally sensitive, and accessible to patients.

Recognizing the limited access to mental health services and high rates of untreated depression, anxiety, and trauma experienced by patients at our sites—a direct result of racism, discrimination, and the inequitable distribution of the social determinants of health—the curriculum committee incorporated evidence-based behavioral health skills that participants could utilize to cope with strong emotions, manage daily frustrations, and reduce stress.

For example, each session includes a mindfulness-based stress-reduction activity, a brief emotion regulation activity, and a self-care activity. The behavioral health activities are repeated throughout the curriculum in various ways to facilitate multiple opportunities for practice and to demonstrate how to apply them to different settings, such as managing labor pain or the first days home in the postpartum period.

EleVATE GC was facilitated by an obstetric clinician (obstetrician, midwife, nurse practitioner, or family practice physician) and cofacilitator (often a medical assistant, health educator, or social worker). A psychologist was also available to help cofacilitate each pilot EleVATE group to better support facilitators in executing the behavioral health components of the curriculum through coaching and feedback. The EleVATE GC facilitator's guide incorporates trauma-informed principles and practices, facilitator notes about trauma awareness, instruction on facilitating conversations about trauma, descriptions of how trauma is connected to physical and mental health topics, and skill-building opportunities for facilitators to implement and practice.

### Training process and associated activities

**FIG. 1.**
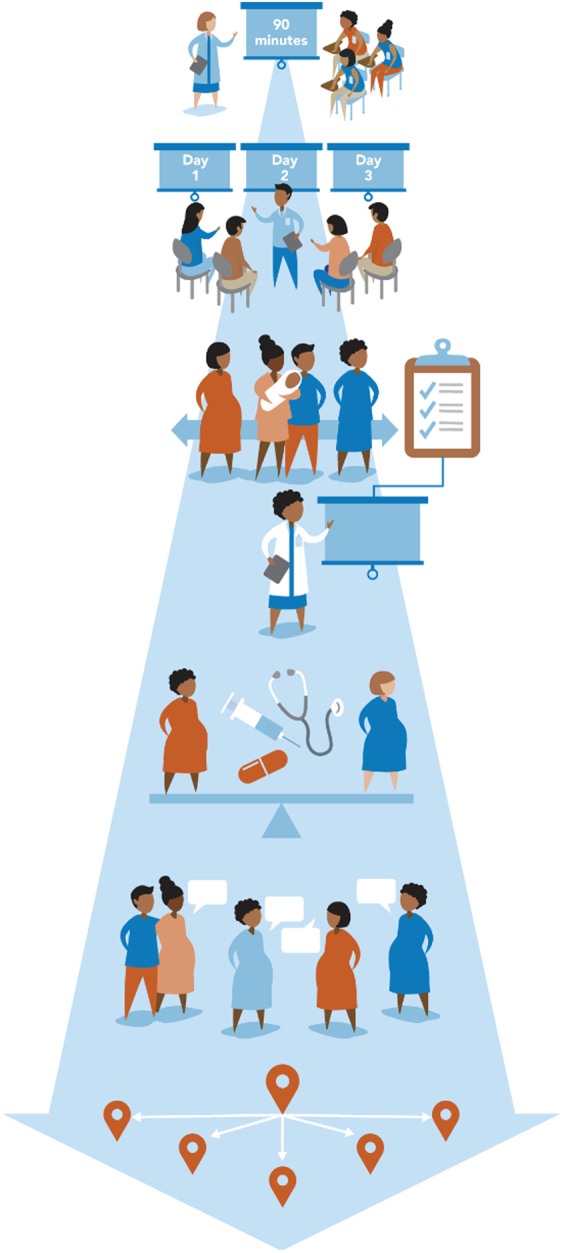
EleVATE training pyramid: EleVATE training occurred in addition to any baseline “cultural competence” or “implicit bias” training at each clinical site. All patient-facing staff and clinical sites received a 90-min trauma-informed care training. In addition, EleVATE facilitators received group facilitation, behavioral health integration, and crossroads anti-racism training. Finally, facilitators had the opportunity to apply these skills through long-term continuity experience facilitating EleVATE with groups of pregnant patients. EleVATE, Elevating Voices, Addressing Depression, Toxic Stress and Equity.

Training EleVATE GC facilitators and the health care teams at their respective clinical sites was a key part of the intervention. Each site had pre-existing cultural competency trainings that ranged from online modules to in-person trainings that were usually completed annually (Fig. 1; Level 1). To support the goals of the EleVATE Collaborative, health care teams (front desk staff, medical assistants, nurses, phlebotomists, clinicians, managers, etc.) from each site participating in the pilot study were invited to a 90-min trauma-informed care training focused on introducing trauma-informed care, ways to implement trauma-informed care practices and patient-focused care (Fig. 1; Level 2).

Community collaborators participated in a separate training on community trauma with a racial equity lens as an introduction to trauma-informed care (Fig. 1; Level 2). Next, community collaborators and health care team members, including all facilitators of group prenatal care at EleVATE sites, attended a 3-day trauma-informed care and racial equity intensive training (Fig. 1; Level 3). These trainings were conducted by *Alive and Well Communities*, a local nonprofit organization dedicated to reducing the impact of trauma on health and well-being.

After the curriculum team finished the curriculum, it was distributed to facilitators who then participated in a 1-day training in the EleVATE group prenatal care model (Fig. 1; Level 4). Finally, EleVATE GC facilitators participated in additional training on antioppressive principles and dismantling racism, conducted by *Crossroads Antiracism Organizing and Training*, an organization specializing in antiracism capacity building and technical assistance (Fig. 1; Level 5).

### Phase II: Pilot study

The evaluation committee, in partnership with community collaborators, identified the outcomes most meaningful to pregnant people impacted by racial inequities, which were the association between the rates of preterm birth, small for gestational age, and perinatal depression between patients participating in the group interventions compared with IC. The goal of the pilot study was to determine feasibility of the EleVATE GC intervention.

### Setting and participants

Three organizations participated in the EleVATE GC pilot study: Affinia Healthcare (three sites), which is the largest Federally Qualified Health Center (FQHC) in St. Louis, and two academic hospital-based obstetrics and gynecology clinic sites at Barnes-Jewish Hospital (Washington University School of Medicine) and St. Mary's Hospital (St. Louis University School of Medicine). Participants in this pilot study were a convenience sample of patients presenting for initial prenatal care at a participating site who chose to participate in one of three care modalities: IC, CP, and EleVATE GC.

Inclusion criteria were pregnant patients who were ≥13 years of age with a singleton pregnancy. Patients were not eligible if they had a known fetal anomaly, or if the obstetric facilitator determined that a serious medical or psychiatric comorbidity including psychosis, mania, substance use disorders, or suicidal ideation precluded group care. At the FQHC and one of the hospital-based clinics, participants were prospectively enrolled in the study. Research staff approached eligible patients about participation and interested patients provided written informed consent. At the other hospital-based clinic, the data were collected as part of an ongoing quality assurance project.

This study, including a waiver of consent to use the quality assurance data, was approved by the institutional review board at Washington University School of Medicine. The research was completed in accordance with the Declaration of Helsinski as revised in 2013.

### Study design

All participants completed baseline surveys at enrollment (initial study visit), their last prenatal visit before delivery, and between 4 and 12 weeks postpartum. Patients delivering unexpectedly early completed the second set of surveys soon after delivery. Depressive symptoms were measured using the Edinburgh Postnatal Depression Scale (EPDS),^31^ a 10-item scale assessing depressive symptoms validated for use in both the prenatal and postpartum periods.^37,38^ We also collected data on additional psychosocial measures of stress, anxiety, and trauma (see Supplementary Data).

Study staff reviewed electronic health records and abstracted participant data including demographics, medical and surgical history, prenatal laboratory results, delivery information, and the postpartum course. The primary outcomes were preterm birth (delivery at <37 weeks gestation), small for gestational age (birthweight <10th percentile on Alexander growth curve^39^), and postpartum positive depression screen (EPDS >10).

### Data analyses

We used Research Electronic Data Capture (REDCap) for data collection and management.^40,41^ We calculated values for missing items using mean imputation as appropriate. We conducted two sets of comparisons: participants receiving EleVATE GC versus IC, and participants receiving group care (EleVATE GC and CP) versus IC. This pilot study was designed as a descriptive feasibility study and was not statistically powered to test for significance.

## Results

Forty-eight people who all self-identified as women were enrolled in the study (*n*=11 IC; *n*=14 CP; *n*=23 EleVATE GC): 43 African American (86%), 3 White (6%), 1 Latina (2%), and 3 who identified as another race (6%). Baseline demographics were similar between groups and are displayed in Table 2. Prenatal care attendance was similar between those in IC versus the group care models (IC 8.6 visits ±2.7; CP 11.4 visits ±4.3; EleVATE GC 9.4 visits ±4.3).

**Table 2.** Participant Characteristics Stratified by Type of Prenatal Care

		EleVATE versus IC		Group versus IC	
	IC (***n***=11)	EleVATE GC (***n***=23)	***p***	All group care (***N***=37)	***p***
Maternal age	25.0±6.5	23.3±3.5	0.33	22.7±0.7	0.18
Race/ethnicity			0.21		0.10
Black	8 (72.7)	21 (91.3)		35 (94.6)	
White	2 (18.2)	1 (4.4)		1 (2.7)	
Hispanic	1 (9.1)	1 (4.4)		1 (2.7)	
Insurance			1.00		1.00
Medicaid	9 (81.8)	19 (82.6)		30 (83.3)	
Commercial	2 (18.2)	4 (17.4)		5 (13.9)	
Disability	0 (0.0)	0 (0.0)		1 (2.8)	
Nulliparous	7 (63.6)	13 (56.5)	1.00	22 (59.5)	1.00
History of preterm birth	0 (0.0)	1 (4.4)	1.00	2 (5.4)	1.00
History of cesarean section	0 (0.0)	0 (0.0)	—	1 (2.7)	1.00
Medical comorbidities					
Asthma	3 (27.3)	7 (30.4)	1.00	14 (37.8)	0.72
Chronic hypertension	0 (0.0)	1 (4.4)	1.00	2 (5.4)	1.00
Mental health diagnosis	3 (27.3)	5 (21.7)	1.00	7 (18.9)	0.68
Alcohol use	1 (10.0)	0 (0.0)	0.30	2 (5.4)	0.52
Tobacco use	1 (9.1)	3 (13.0)	1.00	5 (13.5)	1.00
Marijuana use	1 (10.0)	1 (4.4)	0.52	1 (2.7)	0.38

Data are presented as mean±SD, *n* (%) and median (interquartile range); missing values were not included in column percentages or bivariate analyses; differences were assessed using Student's *t*-test, chi-square, Fisher's exact test, and Wilcoxon rank-sum as appropriate.

EleVATE, Elevating Voices, Addressing Depression, Toxic Stress and Equity; GC, group prenatal care; IC, individual care.

Gestational ages at delivery and preterm birth <37 weeks were similar across the three groups (IC 18.2%, EleVATE GC 0%, CP 7.1%; Table 3 and Supplementary Table S3). There were two (18.2%) preterm births <34 weeks in the IC group and 0 in the EleVATE GC and CP groups (Table 3 and Supplementary Table S3). Patients participating in group prenatal care (EleVATE GC or CP) were significantly less likely to experience an early preterm birth <34 weeks (IC 18.2%, group care 0.0%; IC vs. group care *p*=0.05; Table 3).

**Table 3.** Maternal and Neonatal Pregnancy Outcomes

		EleVATE versus IC		Group versus IC	
	IC (***n***=11)	EleVATE GC (***n***=23)	***p***	All group care **(*N***=37)	***p***
Primary outcome					
Gestational age at delivery, weeks	39.4 (37.6–40.3)	39.2 (38.3–40.1)	0.91	39.2 (38.2–40.0)	0.96
Preterm birth <37 weeks	2 (18.2)	0 (0.0)	0.10	1 (2.7)	0.13
Preterm birth <34 weeks	2 (18.2)	0 (0.0)	0.10	0 (0.0)	0.05
Pregnancy outcomes					
Number of study visits attended	8.6±2.7	9.4±4.3	0.54	10.2±0.7	0.25
Hypertensive disorder of pregnancy	1 (9.1)	6 (26.1)	0.38	9 (24.3)	0.42
Cesarean section	3 (27.3)	5 (21.7)	1.00	6 (16.2)	0.41
Small for gestational age	0 (0.0)	2 (9.1)	1.00	3 (8.6)	1.00
Special care/NICU admission	4 (36.4)	3 (13.0)	0.18	4 (10.8)	0.07
Breastfeeding at discharge	6 (54.6)	18 (78.3)	0.16	29 (80.6)	0.08
Six-week postpartum outcomes					
Attended postpartum visit	6 (54.6)	19 (82.6)	0.11	31 (83.8)	0.04
Breastfeeding	1 (16.7)	10 (62.5)	0.15	18 (69.2)	0.03
Contraceptive method initiated by 6 weeks	7 (100.0)	17 (89.5)	1.00	27 (90.0)	1.00
Edinburgh Depression Scale					
Baseline	4 (2–9)	5 (2–7)	0.84	6 (3–9)	0.66
Delivery	8 (4–11)	5 (1–9)	0.20	5 (1–10)	0.29

Data are presented as mean±SD, median (interquartile range), and *n* (%); Differences were assessed using Student's *t*-test, chi-square, and Fisher's exact test as appropriate.

SD, standard deviation.

There were no differences in SGA, perinatal depression (baseline, delivery), or other pregnancy outcomes (Table 3). At 6 weeks postpartum, participants in group care were more likely to attend their postpartum visit (IC 54.6%, EleVATE GC 82.6%, CP 83.8%; IC vs. group care *p*=0.04) and breastfeed at hospital discharge (IC 16.7%, EleVATE GC 62.5%, CP 69.2%; IC vs. group care *p*=0.03). The difference in perinatal depression scores at postpartum could not be interpreted due to the amount of missing data: 36% IC, 25% EleVATE GC, 32% all group care.

## Discussion

Addressing the overwhelming disparities evident in pregnancy health and infant mortality is essential. The EleVATE Collaborative worked to engage multiple institutions and align numerous health services and resources crucial to patient well-being and improving population health. Our results suggest that EleVATE GC is a promising and feasible approach to group prenatal care with potential to reduce racial disparities in pregnancy outcomes.

It is key to leverage the prenatal period, providing opportunities for mental health skill building and addressing inequitable adverse pregnancy outcomes for infants and pregnant people by training health care teams and community collaborators in antiracist practices, trauma-informed care, behavioral health, and health equity. The content, implementation, and evaluation of EleVATE GC were created through strong patient leadership and CBPR methods.

In addition to increasing patients' self-efficacy and engagement in care, we hypothesize that the EleVATE training, coupled with a shared >20-h experience with a group of patients who are often socially dissimilar, increases clinician's empathy and mitigates the impact of implicit bias and racism on patient care. Additional research is needed beyond this descriptive feasibility study to determine whether EleVATE is effective in improving pregnancy outcomes and promoting health equity. Future studies will also be needed to test the comparative effectiveness of EleVATE GC to other group care models such as CP.

The extensive training we provided to community collaborators, health care staff, and facilitators may impact the feasibility and generalizability of implementing EleVATE GC. If EleVATE GC proves effective, we must determine the potential mechanisms by which this happens and the best ways to support health care teams to implement the intervention.

This pilot study had several strengths, including a long-term community–academic partnership led by patients who actively cocreated the intervention, set priorities for evaluation, and served on the steering committee of the collaborative. Although participating clinics were all safety-net providers in the region, there was diversity with inclusion of large academic centers and three FQHC sites throughout the St. Louis community.

Our findings should be interpreted in the context of the following limitations. We were not powered to see differences in clinical outcomes. Although patients in group care were less likely to experience an early preterm birth in unadjusted analysis, it remains possible that this difference is a Type I error. This was a small convenience sample and prone to selection bias, since patients who choose to participate in group care may have other characteristics that influence pregnancy outcomes, despite similar demographic characteristics.

Furthermore, patients who may have benefitted from EleVATE group care may have chosen to participate in another form of care. Since our goal was to train obstetric clinicians to be mental health extenders, a mental health specialist was available to cofacilitate the groups, which may not be generalizable to other settings. Finally, there was a high level of missing postpartum data from one site; thus, the postpartum findings are prone to bias.

## Implications for Health Equity

EleVATE GC demonstrates promising trends to address health inequities through our pilot study and collaborative process. A large randomized hybrid implementation–effectiveness trial is now underway to further test this new model of prenatal care and the impact that it may have on both patients and clinicians.

## Supplementary Material

Supplemental data

Supplemental data

Supplemental data

Supplemental data

## Abbreviations Used

CBPR: community-based participatory research
CP: CenteringPregnancy™
EleVATE: Elevating Voices, Addressing Depression, Toxic Stress and Equity
EPDS: Edinburgh Postnatal Depression Scale
FQHC: Federally Qualified Health Center
GC: group prenatal care
IC: individual care
IHN: Integrated Health Network
SD: standard deviation
