# Supplementary material for: Elevating Voices, Addressing Depression, Toxic Stress, and Equity Through Group Prenatal Care: A Pilot Study
Source: Health Equity. 2024 Jan 29;8(1):87–95. doi: 10.1089/heq.2023.0160 (PMC10823176; doi:10.1089/heq.2023.0160)
Supplement: Supplemental data [file Suppl_TableS3.docx]

Supplemental Table 3: Maternal and Neonatal Outcomes stratified by type of group prenatal care

|  | EleVATE GC  n=23 | Centering Pregnancy  n=14 | *p* |
| --- | --- | --- | --- |
| *Primary Outcome* |  |  |  |
| Gestational age at delivery, wks | 39.2 (38.3-40.1) | 38.9 (37.6-40.0) | 0.52 |
| Preterm birth <37 weeks | 0 (0.0) | 1 (7.1) | 0.38 |
| Preterm birth <34 weeks | 0 (0.0) | 0 (0.0) | -- |
| *Pregnancy Outcomes* |  |  |  |
| Number of study visits attended | 9.4 ± 4.3 | 11.4 ± 4.3 | 0.20 |
| Hypertensive disorder of pregnancy | 6 (26.1) | 3 (21.4) | 1.00 |
| Cesarean section | 5 (21.7) | 1 (7.1) | 0.38 |
| Small for gestational age | 2 (9.1) | 1 (7.7) | 1.00 |
| *Missing* | *1* | *1* |  |
| Special care/NICU admission | 3 (13.0) | 1 (7.1) | 1.00 |
| Breastfeeding at discharge | 18 (78.3) | 11 (84.6) | 1.00 |
| *Missing* | *0* | *1* |  |
| *Six Week Postpartum Outcomes* |  |  |  |
| Attended postpartum visit | 19 (82.6) | 12 (85.7) | 1.00 |
| Breastfeeding | 10 (62.5) | 8 (80.0) | 0.42 |
| *Missing* | *7* | *4* |  |
| Contraceptive method initiated by 6wks | 17 (89.5) | 10 (90.9) | 1.00 |
| *Missing* | *4* | *3* |  |
| *Edinburgh Postnatal Depression Scale* | | | |
| Baseline | 5 (2-7) | 8 (4-10) | **0.04** |
| *Missing* | *0* | *0* |  |
| Delivery | 5 (1-9) | 7 (1-11) | 0.56 |
| *Missing* | *1* | *1* |  |
| Data are presented as mean ± SD and n (%)  Differences were assessed using Student’s t-test, chi-square, and Fisher’s exact as appropriate | | | |
