# Supplementary material for: Elevating Voices, Addressing Depression, Toxic Stress, and Equity Through Group Prenatal Care: A Pilot Study
Source: Health Equity. 2024 Jan 29;8(1):87–95. doi: 10.1089/heq.2023.0160 (PMC10823176; doi:10.1089/heq.2023.0160)
Supplement: Supplemental data [file Suppl_TableS1.docx]

Supplemental Table 1: Perinatal mental health measures completed by patients at enrollment into the study, last prenatal visit prior to delivery, and 6 weeks postpartum

|  |  | EleVATE vs Individual Care | | Group vs Individual Care | |
| --- | --- | --- | --- | --- | --- |
| Scores | Individual Care  n=11 | EleVATE GC  n=23 | *p* | All Group Care  N=37 | *p* |
| *Perceived Stress Scale* | | |  |  |  |
| Baseline | 16 (11-17) | 12 (7-20) | 0.24 | 13 (10-20) | 0.88 |
| Delivery | 11 (8-18) | 12 (6-15) | 0.46 | 12 (5-16) | 0.51 |
| Postpartum | 12 (7-18) | 7 (1-14) | 0.26 | 9 (5-15) | 0.54 |
| *Missing* | 4 | 8 |  | 12 |  |
| *Generalized Anxiety Disorder-7* | | |  |  |  |
| Baseline | 5 (2-9) | 2 (0-9) | 0.18 | 4 (0-9) | 0.36 |
| Delivery | 5 (1-8) | 2 (0-5) | 0.20 | 3 (0-9) | 0.38 |
| Postpartum | 4 (0-12) | 0 (0-1) | **0.04** | 1 (0-7) | 0.29 |
| *Missing* | 4 | 8 |  | 12 |  |
| *Post-Traumatic Stress Disorder Checklist-5* | | |  |  |  |
| Baseline | 15 (9-24) | 5 (1-21) | 0.11 | 8 (3-25) | 0.29 |
| Delivery | 9 (3-14) | 6 (0-13) | 0.50 | 7 (0-19) | 0.83 |
| Postpartum | 4 (1-24) | 5 (0-9) | 0.50 | 6 (1-15) | 0.89 |
| *Missing* | 4 | 8 |  | 12 |  |
| Data are presented as median (interquartile range) due to non-normal distribution of scores  Differences were assessed using Wilcoxon rank-sum | | | | | |
