# Supplementary material for: Elevating Voices, Addressing Depression, Toxic Stress, and Equity Through Group Prenatal Care: A Pilot Study
Source: Health Equity. 2024 Jan 29;8(1):87–95. doi: 10.1089/heq.2023.0160 (PMC10823176; doi:10.1089/heq.2023.0160)
Supplement: Supplemental data [file Suppl_TableS2.docx]

Supplemental Table 2: Participant characteristics stratified by type of group prenatal care

|  | EleVATE GC  n=23 | Centering Pregnancy  n=14 | *p* |
| --- | --- | --- | --- |
| Maternal age | 23.3 ± 3.5 | 21.8 ± 5.4 | 0.31 |
| Race/ethnicity |  |  | 1.00 |
| Black | 21 (91.3) | 14 (100.0) |  |
| White | 1 (4.4) | 0 (0.0) |  |
| Hispanic | 1 (4.4) | 0 (0.0) |  |
| Insurance |  |  | 0.32 |
| Medicaid | 19 (82.6) | 11 (84.6) |  |
| Commercial | 4 (17.4) | 1 (7.7) |  |
| Disability | 0 (0.0) | 1 (7.7) |  |
| *Missing* | *0* | *1* |  |
| Nulliparous | 13 (56.5) | 9 (64.3) | 0.74 |
| History of preterm birth | 1 (4.4) | 1 (7.1) | 1.00 |
| History of cesarean section | 0 (0.0) | 1 (7.1) | 0.38 |
| Medical comorbidities |  |  |  |
| Asthma | 7 (30.4) | 7 (50.0) | 0.30 |
| Chronic hypertension | 1 (4.4) | 1 (7.1) | 1.00 |
| Mental health diagnosis | 5 (21.7) | 2 (14.3) | 0.69 |
| Alcohol use | 0 (0.0) | 2 (14.3) | 0.14 |
| Tobacco use | 3 (13.0) | 2 (14.3) | 1.00 |
| Marijuana use | 1 (4.4) | 0 (0.0) | 1.00 |
| Data are presented as mean ± SD, n (%) and median (interquartile range); missing values were not included in column percentages or bivariate analyses  Differences were assessed using Student’s t-test, chi-square, Fisher’s exact, and Wilcoxon rank-sum as appropriate | | | |
