# Supplementary material for: Elevating Voices, Addressing Depression, Toxic Stress, and Equity Through Group Prenatal Care: A Pilot Study
Source: Health Equity. 2024 Jan 29;8(1):87–95. doi: 10.1089/heq.2023.0160 (PMC10823176; doi:10.1089/heq.2023.0160)
Supplement: Supplemental data [file Suppl_TableS4.docx]

Supplemental Table 4: Perinatal mental health measures completed by patients at enrollment into the study, last prenatal visit prior to delivery, and 6 weeks postpartum stratified by type of group prenatal care

| Scores | EleVATE GC  n=23 | Centering Pregnancy  n=14 | *p* |
| --- | --- | --- | --- |
| *Perceived Stress Scale* | | |  |
| Baseline | 12 (7-20) | 20 (13-23) | **0.01** |
| *Missing* | 0 | 0 |  |
| Delivery | 12 (6-15) | 11 (4-21) | 0.80 |
| *Missing* | 1 | 1 |  |
| Postpartum | 7 (1-14) | 12 (7-23) | 0.19 |
| *Missing* | 8 | 4 |  |
| *Generalized Anxiety Disorder-7* | | |  |
| Baseline | 2 (0-9) | 5 (1-10) | 0.29 |
| *Missing* | 0 | 1 |  |
| Delivery | 2 (0-5) | 4 (2-13) | 0.18 |
| *Missing* | 1 | 1 |  |
| Postpartum | 0 (0-1) | 8 (0-13) | **0.02** |
| *Missing* | 8 | 4 |  |
| *Post-Traumatic Stress Disorder Checklist-5* | | |  |
| Baseline | 5 (1-21) | 16 (6-29) | 0.05 |
| *Missing* | 0 | 1 |  |
| Delivery | 6 (0-13) | 10 (3-19) | 0.25 |
| *Missing* | 1 | 1 |  |
| Postpartum | 5 (0-9) | 22 (1-29) | 0.12 |
| *Missing* | 8 | 4 |  |
| Data are presented as median (interquartile range) due to non-normal distribution of scores  Differences were assessed using Wilcoxon rank-sum | | | |
